# Are we over-treating with checkpoint inhibitors?

**DOI:** 10.1038/s41416-019-0570-y

**Published:** 2019-09-17

**Authors:** Sarah Danson, Jane Hook, Helen Marshall, Alexandra Smith, Sue Bell, Simon Rodwell, Pippa Corrie

**Affiliations:** 1 University of Sheffield and Sheffield Teaching Hospitals NHS Foundation Trust, Sheffield, UK; 2 Leeds CTRU, University of Leeds, Leeds, UK; 3 Melanoma Focus, Cambridge, UK; 4 Cambridge University Hospitals NHS Foundation Trust and University of Cambridge, Cambridge, UK

**Keywords:** Melanoma, Immunotherapy

## Abstract

Anti-PD-1 antibodies offer potentially life-saving treatment for some cancer patients, but their chronic administration generates high and ever-increasing costs. Despite licensing for long-term use, optimal treatment duration is unknown. We challenge the need for long-term treatment duration, using evidence from melanoma research, both published and in process.

## Main

Anti-PD-1 antibodies (pembrolizumab and nivolumab) are licensed to be administered to metastatic melanoma patients until disease progression. Multiple anti-PD(L)-1 antibodies are being licensed to treat various different cancer types, all with prolonged durations of treatment to 2 years or more for responding patients. The question of whether anti-PD(L)-1 therapy needs administering continuously to generate an immune response is taxing the global oncology community. Chronic administration generates a significant burden for patients and healthcare systems, entailing multiple clinic visits and the risk of chronic, life changing and sometimes life-threatening immune-mediated toxicities. The health-economic impact is substantial, which not all healthcare systems can absorb.^1^

The first checkpoint inhibitor to enter the clinic was the anti-CTLA-4 antibody ipilimumab. In contrast to anti-PD(L)-1 antibodies, ipilimumab is administered over 12 weeks only, and around 20% of patients will sustain durable remissions in the absence of ongoing infusions. CTLA-4 and PD-1 differ in their T-cell receptor role and function, yet there is no biological evidence justifying continuous therapy with anti-PD(L)-1 antibodies.^2^ Indeed, contrary evidence is now accumulating.

Long-term follow-up of metastatic melanoma patients treated in the first prospective trials evaluating anti-PD-1 suggests treatment to progression may not be justified.^2–4^ In the Keynote-001 pembrolizumab trial, 105 of 655 (17%) recruited patients had a complete response and 67 of 105 stopped pembrolizumab while still in complete response, mostly due to patient choice. The 2-year disease-free survival rate from the time of complete response was 90% for all, whether or not they stopped treatment.^5^ In the Keynote-006 trial comparing pembrolizumab with ipilimumab as first line immunotherapy for metastatic melanoma, the planned treatment with pembrolizumab was 2 years.^6^ A total of 104 of 556 (19%) patients completed the planned course. After following the 104 patients for a median of 9 months, their progression-free survival (PFS) was 91%: 95% for complete responders, 91% for partial responders, and 83% for those with stable disease. A total of 17% of patients experienced severe (grade 3/4) toxicity during treatment. Based on these data, many clinicians and patients are electing to stop treatment at 2 years.^3,6^

For metastatic melanoma, 40% of patients can expect to respond to anti-PD-1 antibodies and therefore are likely to be eligible to continue treatment to 2 years or more.^3,4^ Most responses to anti-PD-1 antibodies occur within 6 months of starting treatment and there is growing motivation to stop treatment before 2 years.^7^ A recent retrospective study determined that ‘real-life’ duration of treatment is shorter than that reported in clinical trials; patients with a complete response (CR) compared with a partial response (PR) or stable disease may have a lower risk of relapse off therapy. In those with CR, the risk of progression was significantly higher in those treated for <6 months compared with those treated for >6 months.^8^ Another retrospective review of 104 progression-free metastatic melanoma patients undergoing FDG-PET/CT after 1 year of anti-PD-1 antibodies reported that complete metabolic response (CMR) was associated with 2-year PFS of 96%, compared with 49% in those patients whose scans did not show CMR (HR [hazard ratio] 0.06, 95% CI [confidence interval] 0.02–0.23), so other tools may offer value in tailoring treatment in the future.^9^ Even so, national reimbursement models are generally licence-driven and neither stopping early, nor treatment re-challenge, may actually be permitted. There is clearly a need to generate high-quality evidence to define early stopping rules.

The CheckMate153 study is the only randomised study published to date specifically evaluating duration of anti-PD-1 therapy. CheckMate153 compared treatment until progression with 12 months of nivolumab in patients with advanced non-small cell lung cancer (NSCLC). In this study, 220 patients receiving nivolumab who were progression-free at 12 months were randomised to continue until progression, or to stop treatment; patients in the discontinuation arm were allowed to re-start nivolumab at progression. Initial results^10^ reported better PFS with continuous versus 12-months treatment: median PFS was not reached in the continuous arm compared with 10.3 months (95% CI 6.4–15.2) in the discontinuation arm (HR 0.42, 95% CI 0.25–0.71). Despite PFS differences, overall survival did not show a statistically significant difference between the two treatment arms (HR 0.63, 95% CI 0.33–1.20), although the data are immature. Whether these results are generalisable to other tumour types needs to be determined and two key prospective clinical trials are now under way in metastatic melanoma. Both are pragmatic and use standard of care, government-funded anti-PD-1 therapy.

The Canadian STOP-GAP study (NCT02821013) is currently assessing intermittent versus continuous treatment with anti-PD-1 inhibitors, with a primary endpoint of overall survival. In this trial, 614 patients are being randomised in the first 16 weeks of anti-PD-1 treatment to either standard 2 years of treatment or treatment to maximal tumour response with retreatment at the time of progression. Maximal tumour response is determined by at least two radiological measurements 3 months apart. STOP-GAP therefore is primarily evaluating the role of re-challenge rather than the specific question of optimal treatment duration.

The UK National Institute for Health Research (NIHR) portfolio DANTE trial (ISRCTN15837212) is randomising metastatic melanoma patients receiving anti-PD-1 therapy who are progression-free at 12 months to either stop (with re-challenge allowed on progression) or continue treatment as per standard use. This non-inferiority trial has a primary endpoint of PFS. Patients are being registered in the first year of treatment, with a plan to randomise 1208 patients at 12 months. Opportunities are being explored to utilise this unique trial to evaluate biomarkers of treatment response as well as toxicity, including genetic signatures, circulating tumour DNA, gut microbiota and ^18^F-FDG PET/CT.

We encourage patients and investigators alike to support prospective randomised controlled trials like DANTE and STOP-GAP, generating the evidence needed to ensure safe, effective and affordable treatment for our patients now and in the future.

## Data Availability

Not applicable

## References

[1] da Veiga CRP, da Veiga CP, Drummond-Lage AP (2018). Concern over cost of and access to cancer treatments: a meta-narrative review of nivolumab and pembrolizumab studies. Crit. Rev. Oncol. Hematol..

[2] Hamid O, Robert C, Daud A, Hodi FS, Hwu WJ, Kefford R (2019). Five-year survival outcomes for patients with advanced melanoma treated with pembrolizumab in KEYNOTE-001. Ann. Oncol..

[3] Robert C, Schachter J, Long GV, Arance A, Grob JJ, Mortier L (2015). Pembrolizumab versus ipilimumab in advanced melanoma. N. Engl. J. Med.

[4] Wolchok JD, Chiarion-Sileni V, Gonzalez R, Rutkowski P, Grob JJ, Cowey CL (2017). Overall survival with combined nivolumab and ipilimumab in advanced melanoma. N. Engl. J. Med.

[5] Robert C, Ribas A, Hamid O, Daud A, Wolchok JD, Joshua AM (2018). Durable complete response after discontinuation of pembrolizumab in patients with metastatic melanoma. J. Clin. Oncol..

[6] Schachter J, Ribas A, Long GV, Arance A, Grob JJ, Mortier L (2017). Pembrolizumab versus ipilimumab for advanced melanoma: final overall survival results of a multicenter, randomised, open-label phase 3 study (KEYNOTE-006). Lancet.

[7] Khushalani NI (2018). Duration of anti-programmed death-1 therapy in advanced melanoma: how much of a good thing is enough?. J. Clin. Oncol..

[8] Jansen YJL, Rozeman EA, Mason R, Goldringer SM, Geukes Foppen MH, Hoejbergs L (2019). Discontinuation of anti-PD-1 antibody therapy in the absence of disease progression or treatment limiting toxicity: clinical outcomes in advanced melanoma. Ann. Oncol..

[9] Tan AC, Emmett L, Lo S, Liu V, Kapoor R, Carlino MS (2018). FDG-PET response and outcome from anti-PD-1 therapy in metastatic melanoma. Ann. Oncol..

[10] Spigel D., McCleod M., Hussein M., Waterhouse D. M., Einhorn L., Horn L., et al. Randomized results of fixed-duration (1-yr) vs continuous nivolumab in patients (pts) with advanced non-small cell lung cancer (NSCLC). *Ann. Oncol.* 2017; **28**, 461 (Suppl 5), abstract 12970 (2017).

